# The Critical Role of Thioacetamide Concentration in the Formation of ZnO/ZnS Heterostructures by Sol-Gel Process

**DOI:** 10.3390/nano8020055

**Published:** 2018-01-23

**Authors:** Eloísa Berbel Manaia, Renata Cristina Kiatkoski Kaminski, Bruno Leonardo Caetano, Marina Magnani, Florian Meneau, Amélie Rochet, Celso Valentim Santilli, Valérie Briois, Claudie Bourgaux, Leila Aparecida Chiavacci

**Affiliations:** 1 Department of Drugs and Medicines, School of Pharmaceutical Sciences, São Paulo State University (UNESP), Araraquara, São Paulo 14800-903, Brazil; caetano@fcfar.unesp.br; 2 Institut Galien, University Paris-Sud, The National Center for Scientific Research (CNRS), UMR 8612, 92296 Châtenay-Malabry, France; claudie.bourgaux@u-psud.fr; 3 Department of Chemistry, Sergipe Federal University—Campus Itabaiana, Av. Vereador Olimpio Grande, s/n—Itabaiana, SE 49506-036, Brazil; re_kaminski@hotmail.com; 4 Chemistry Institute of São Paulo State University—UNESP, Prof. Francisco Degni Street, 55, Araraquara, São Paulo 14800-060, Brazil; marina@iq.unesp.br (M.M.); santilli@iq.unesp.br (C.V.S.); 5 Brazilian Synchrotron Light Laboratory (LNLS), Brazilian Center for Research in Energy and Materials (CNPEM), São Paulo 13083-970, Brazil; florian.meneau@lnls.br (F.M.); amelie.rochet@lnls.br (A.R.); 6 Synchrotron Optimized Light Source of Intermediate Energy to LURE (SOLEIL), L’Orme des Merisiers, BP48, Saint Aubin, 91192 Gif-sur Yvette, France; valerie.briois@synchrotron-soleil.fr

**Keywords:** ZnO, ZnS, quantum dots, heterostructure, sol-gel, Small Angle X-ray Scattering, X-ray Absorption Spectroscopy, UV-vis spectroscopy

## Abstract

ZnO/ZnS heterostructures have emerged as an attractive approach for tailoring the properties of particles comprising these semiconductors. They can be synthesized using low temperature sol-gel routes. The present work yields insight into the mechanisms involved in the formation of ZnO/ZnS nanostructures. ZnO colloidal suspensions, prepared by hydrolysis and condensation of a Zn acetate precursor solution, were allowed to react with an ethanolic thioacetamide solution (TAA) as sulfur source. The reactions were monitored in situ by Small Angle X-ray Scattering (SAXS) and UV-vis spectroscopy, and the final colloidal suspensions were characterized by High Resolution Transmission Electron Microscopy (HRTEM). The powders extracted at the end of the reactions were analyzed by X-ray Absorption spectroscopy (XAS) and X-ray diffraction (XRD). Depending on TAA concentration, different nanostructures were revealed. ZnO and ZnS phases were mainly obtained at low and high TAA concentrations, respectively. At intermediate TAA concentrations, we evidenced the formation of ZnO/ZnS heterostructures. ZnS formation could take place via direct crystal growth involving Zn ions remaining in solution and S ions provided by TAA and/or chemical conversion of ZnO to ZnS. The combination of all the characterization techniques was crucial to elucidate the reaction steps and the nature of the final products.

## 1. Introduction

ZnO and ZnS are wide band gap semiconductors with outstanding electronic and optical properties. They are used in a wide range of applications such as photocatalysts, sensors, electroluminescent devices and lasers [1,2,3,4]. More specifically, ZnO quantum dots (QDs) are promising luminescent probes for bioimaging due to their biodegradability and very low toxicity in vivo [5,6,7,8,9]. In recent years, ZnO/ZnS heterostructures have emerged as an attractive approach for tailoring the particle characteristics and properties [10,11]. When core–shell nanoparticles are formed, the shell acts as a barrier between the core and the surrounding medium, and can change the chemical reactivity and colloidal stability of the core. It is also a strategy for improving the photoluminescence properties of semiconductor nanoparticles (NPs) [12]. Coating NPs with a higher band gap semiconductor can passivate the core surface, eliminating surface-related defect states that induce non-radiative recombination of photogenerated electron–hole pairs (excitons), thereby lowering fluorescence quantum yield and giving rise to “blinking” [13]. ZnO/ZnS core–shell nanocables [14], nanorods [15,16], nanotubes [17], nanowires [18] and nanospheres or powders [19,20,21,22,23], with dimensions in the 25–200 nm range, were obtained. They were synthesized in solution at moderate temperatures via a partial chemical conversion of ZnO to ZnS in the presence of a sulfur source or via addition of both a sulfur source and a Zn precursor to preformed ZnO NPs. Most reports deal with sulfidation processes performed in an aqueous medium using Na_2_S as the sulfur source. Different kinds of shells were obtained, from porous shells formed of small nanoparticles to compact shells ensuring a full coverage and, accordingly, different luminescent properties of the ZnO/ZnS structures were described. 

The aim of our study is to yield insight into the detailed mechanisms of the heterostructure formation, important for a controllable and reproducible synthesis. We have investigated the formation of ZnO/ZnS heterostructures or the conversion of ZnO QDs to ZnS QDs using a straightforward, low-temperature (60 °C) route. ZnO colloidal suspensions were prepared by a sol-gel process, involving hydrolysis and condensation of a Zn acetate precursor solution, as described in our previous reports [24,25,26]. These suspensions were then allowed to react with an ethanolic thioacetamide (TAA) solution, as sulfur source. TAA concentration was varied to reveal the influence of this parameter on the so-formed particles. The formation of ZnO/ZnS nanostructures was monitored by Small Angle X-ray Scattering (SAXS) and UV-vis spectroscopy techniques. Nanoparticles were further characterized by X-ray diffraction (XRD), X-ray Absorption Spectroscopy (XAS) and High Resolution Transmission Electron Microscopy (HRTEM). Different ZnO/ZnS nanostructures were identified, depending on the synthesis conditions. The combination of all the characterization techniques was crucial to elucidate the reaction steps and the final products.

## 2. Materials and Methods

Zinc acetate dehydrate, ${\mathrm{ZnAc}}_{2}\cdot {2H}_{2}O$ (Qhemis, Indaiatuba, Brazil, 98%), thioacetamide, TAA (Sigma-Aldrich, St. Louis, MO, USA, 99.0%), lithium hydroxide monohydrate, LiOH·H_2_O (Vetec, Speyer, Germany, 98%), ethanol (Qhemis, Indaiatuba, Brazil, 99.5%) and heptane (Synth, Diadema, Brazil, 99%), were used as received, without further purification. Zinc oxide (Alfa Aesar, Haverhill, MA, USA) and zinc sulfide (Prolabo, Fontenay-sous-Bois, France) were used as standard.

### 2.1. Synthesis

ZnO colloidal suspensions (ZnO Susp) were synthesized according to the sol-gel route proposed by Spanhel and Anderson [27]. The Zn_4_O(Ac)_6_ tetrameric precursor (labeled herein ZnAc precursor) was first prepared by refluxing an absolute ethanol solution containing 0.05 M ${\mathrm{ZnAc}}_{2}\cdot {2H}_{2}O$ over a period of ~2 h at 80 °C. The thus obtained transparent precursor solution was stored at ~4 °C before to be used for the ZnO QDs preparation. Hydrolysis and condensation reactions leading to the ZnO colloidal suspensions were done by adding under continuous stirring a LiOH·H_2_O absolute ethanol solution (0.5 M). Reactions were carried out at 40 °C and, after 5 s of stirring, the ZnO colloidal suspension was immediately cooled and stored at ~4 °C to prevent any further nucleation and growth process. A nominal molar ratio of [OH]/[Zn] = 0.5 was used. The thus obtained suspension contained about 20% ZnO QDs and 80% remaining ZnAc precursor [24].

To investigate the sulfidation process, 10 mL of ZnO colloidal suspension were allowed to react with 10 mL of TAA ethanolic solution (as sulfur source) under continuous magnetic stirring at 60 °C for 40 min. The suspension was then cooled and stored at ~4 °C. Different concentrations of TAA were used while the other parameters remained unchanged. The final products prepared with different amounts of TAA (1.5, 5 and 50 mM) were designed as TAA1.5, TAA5 and TAA50, respectively. The corresponding [Zn]/[S] nominal molar ratios were 33.33, 10, and 1, respectively.

A ZnS colloidal suspension was synthesized by mixing 10 mL of ZnAc precursor with 10 mL of TAA 5 mM ethanolic solution under continuous stirring at 60 °C for 10 min. The [Zn]/[S] nominal molar ratio was 10. The suspension was then cooled and stored at ~4 °C. This sample, designed as ZnS QDs, was used for comparison with QDs prepared as described above.

### 2.2. Powder Extraction

The as-synthesized colloidal suspensions (ZnO QDs, TAA1.5, TAA5, TAA50 and ZnS QDs) were mixed with a “nonsolvent” heptane [28] (1:4) to induce the precipitation of the QDs, and then centrifuged at 20 °C for 10 min (10,000 rpm). The supernatant solution was discarded and the washed powder was dried under vacuum at room temperature. This method allows extracting the QDs without modifying their size and structure. The dried powders were characterized by XRD and XAS.

### 2.3. Characterization

#### 2.3.1. X-ray Diffraction (XRD) of powders

XRD analysis of the powders was performed on a Bruker D2 PHASER diffractometer (Karlsruhe, Germany) using the Cu Kα radiation, λ = 1.5418 Å, selected by a curved graphite monochromator and a fixed divergence slit of 1/8 deg. in a Bragg–Brentano configuration. The diffraction patterns were measured in the 2θ range 5–70° by the step counting method (0.1 step and 3 s counting time).

#### 2.3.2. High Resolution Transmission Electron Microscopy (HRTEM)

HRTEM investigations were performed with a Philips microscope model CM 200 (FEI Compay, Hillsboro, OR, USA) operating at 200 keV. A drop of the dilute colloidal suspension of QDs was deposited on a copper grid carbon film and dried. Image analysis was carried out with ImageJ (National Institutes of Health, Bethesda, MD, USA) and Digital Micrograph software (Gatan Inc., Pleasanton, CA, USA) packages.

#### 2.3.3. X-ray Absorption Spectroscopy (XAS)

X-ray absorption experiments were performed at the Spectroscopy Applied to Material Based on Absorption (SAMBA) beamline at the French synchrotron source Optimized Light Source of Intermediate Energy to LURE (SOLEIL) (Saint-Aubin, France). A fixed exit sagitally focusing Si (220) double crystal monochromator was used. The grazing incidence of the white and monochromatic beams on both Pd-coated collimating and focusing mirrors was set at 4 mrad, ensuring an efficient harmonic rejection. The beam size was about 2 mm (H) × 0.5 mm (V) at the sample position. The ionization chambers were filled with a N_2_/He gas mixture and the transmission mode was used to record the data. 

The software packages Athena (Washington, DC, USA) and Artemis (Washington, DC, USA) [29] were used to analyze the XAS data. To calibrate each data set in energy the maximum of the first derivative of the zinc reference foil, recorded simultaneously with the data, was used. A linear background was fitted to the pre-edge region and subtracted from the spectra. A post-edge background using the AUTOBK algorithm was applied with a cutoff Rbkg (distance (in Å) for χ(R) above which the signal is ignored) = 1.15 and *k*-weight = 3 in order to isolate the extended X-ray absorption fine structure (EXAFS) oscillations χ(*k*). Then, the Fourier transformations of EXAFS data were carried out between 3.7 and 12 Å^−1^ using a *k*^3^-weighting Kaiser–Bessel window with a dk (FFT window parameter) = 2 apodization window. 

#### 2.3.4. Small-Angle X-ray Scattering (SAXS)

The SAXS measurements were performed at SAXS1 beamline of the Brazilian Synchrotron Light Laboratory (LNLS, Campinas, Brazil). The beamline was equipped with a monochromator (λ = 1.550 Å), a two-dimensional (2D) detector, Pilatus 300 K, localized at 934.934 mm from the sample to record the scattering intensity I(*q*) as a function of the scattering vector, *q*. The resulting scattering vector, *q*, ranged from 0.11 to 4.15 nm^−1^. Silver behenate standard was used to calibrate the *q* range.

The nucleation and growth of nanoparticles have been followed in situ, injecting the freshly prepared reactional solution into the thermostated sample holder (Campinas, Brazil) which was set to 60 °C. Time-resolved SAXS patterns could be recorded from the beginning of the reaction. The curves were collected within an interval of 1 min. 

Data were normalized taking into account the beam decay, acquisition time and sample transmission. The scattered intensity of the sample holders, and the solvent (ethanol) were subtracted from the total intensity. The analysis of the SAXS data was carried out using the software package SASfit (Villigen, Switzerland) [30].

#### 2.3.5. UV-vis Spectroscopy

The absorption spectra were measured using a Cary Win 4000 UV-vis spectrophotometer (Santa Clara, California, United States) with a cuvette of 1mm optical path. Five aliquots were collected at different times (1, 5, 10, 20 and 40 min) for each reaction (TAA1.5, TAA5 and TAA50) and diluted 8 times in ethanol to be recorded in the linear absorption range. The reaction TAA50, as well as ZnS QDs, was also monitored in situ using a Cary 60 UV-vis (Santa Clara, CA, USA) with an immersion probe with optical path of 2 mm.

All spectra of the QDs colloidal suspensions were recorded between 200 and 400 nm, with a wavelength step of 1 nm, and an average counting time of 0.2 s per point. The UV-vis spectra were corrected from the absorption spectrum of ethanol. The size of ZnO QDs was determined from the absorption spectra using the effective mass model derived by Brus [31].

## 3. Results and Discussion

ZnO/ZnS QDs were prepared via a simple sol-gel synthesis. The base-catalyzed hydrolysis and condensation reactions, leading from the ZnAc precursor to ZnO QDs, were stopped before completion and an ethanolic solution of TAA, used as sulfur source, was then added to the suspension containing both ZnO NPs and remaining ZnAc precursor; sulfur ions released upon TAA hydrolyze could react with Zn ions to form ZnS. Insights into the nature and size of crystallites could be obtained from HRTEM and XRD, the later technique being more representative of the whole sample. XAS was recorded at the Zn K-edge to probe the local environment around Zn atoms and evidence ZnO or ZnS structures hardly detectable by XRD because of their very small size and/or lattice disorder. Finally, SAXS and UV-vis spectroscopy allowed monitoring in situ the formation of nanoparticles through either the nanoparticle size measurement or the ZnO and ZnS excitonic peak growth.

### 3.1. Structural Features of Powders

**Figure 1:**
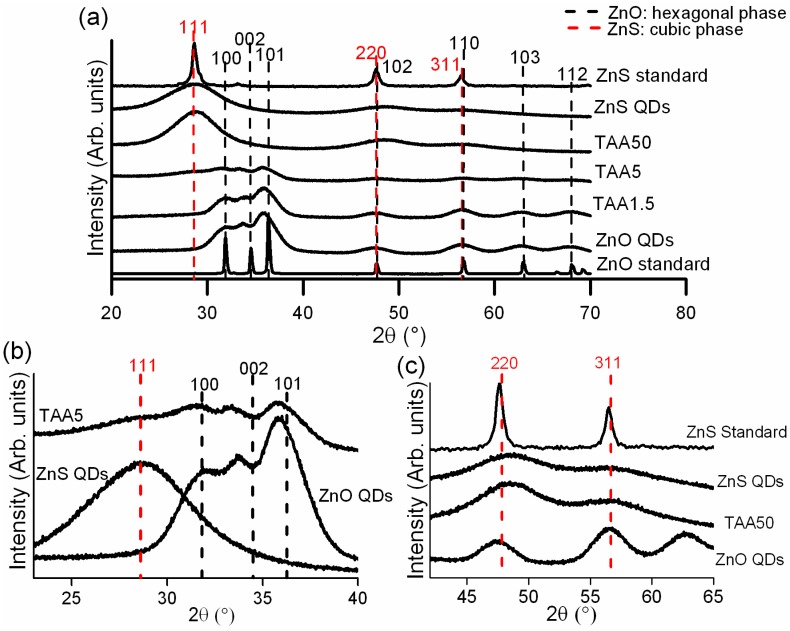
(**a**) X-ray diffraction (XRD) patterns of ZnO and ZnS standards, ZnO and ZnS quantum dots (QDs), and samples prepared with different concentration of the sulfur source (thioacetamide (TAA)), where the vertical lines indicate the hexagonal wurtzite phase (black) and cubic zinc blende phase (red); (**b**) Zoom of the peaks (111), (100), (002) and (101) in the 2θ range from 23° to 40° of the ZnO QDs, ZnS QDs, and TAA5; and (**c**) Zoom of the peaks (220) and (311) in the 2θ range from 42° to 65° of the ZnO QDs, ZnS QDs, ZnS Standard and TAA50.

Diffraction patterns of powders collected at the end of the synthesis are presented in Figure 1a. The positions of peaks characteristic of the ZnO hexagonal wurtzite structure are marked with dashed black lines while those indicative of the ZnS cubic zinc blende structure are marked with dashed red lines. These phases are the most stable for ZnO and ZnS, respectively [32,33]. The TAA1.5 sample exhibits peaks characteristic of ZnO. Figure 1b displays a zoom of the diffraction curves between 2θ = 23° and 2θ = 40° for ZnO QDs, ZnS QDs, and TAA5 sample. A careful observation shows the presence of small broad peaks characteristic of ZnO and ZnS structures in TAA5 sample pattern, pointing out the coexistence of both phases. The TAA50 pattern is similar to that of ZnS QDs, suggesting the formation of the cubic zinc blende structure. However, because of the broadening of the diffraction peaks in the [42°, 65°] 2θ range (Figure 1c), the wurtzite and zinc blende phases could not be distinguished based on XRD patterns only [34]. HRTEM images further supported the formation of the cubic zinc blende structure in TAA50 sample (results not shown).

The average size of ZnO and ZnS nanocrystals could be estimated using the Debye–Scherrer relation [35] applied to the (100), (002) and (101) reflections of ZnO and (111) reflection of ZnS, respectively:

$$D=\frac{k\lambda }{\beta \cos\theta }$$

where *D* is the average crystallite size; k is a constant (shape factor, 0.89 for spherical nanoparticles), λ is the X-ray wavelength, *β* is the Full-Width-at-Half-Maximum (FWHM) of the diffraction peak and 2*θ* is the diffraction angle. The crystallite sizes of ZnO and ZnS QDs were about 5.3 and 1.4 nm, respectively. Regarding TAA5 sample, the crystallite size could not be accurately determined because of the weak broad pattern.

**Figure 2:**
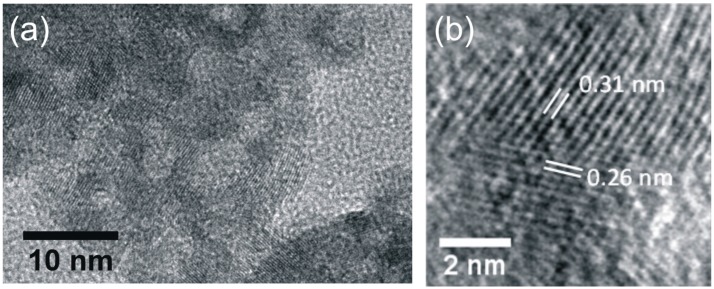
(**a**) High Resolution Transmission Electron Microscopy (HRTEM) image of TAA5 showing in (**b**) the interplanar spacing of ZnO and ZnS phases.

TAA5 sample imaged by HRTEM with low (Figure 2a) and high (Figure 2b) magnification is presented in Figure 2. Figure 2a shows that the sample is crystalline. In Figure 2b, the lattice fringes of two attached ZnO and ZnS crystals are clearly observed. The 0.26 nm spacing arises from the 002 lattice planes of the wurtzite ZnO phase [36], while the 0.31 nm spacing results from the (111) planes of the blende ZnS phase [37]. The HRTEM study corroborates XRD data, revealing the presence of wurtzite ZnO and zinc blende ZnS phases; the morphology evidences the coexistence of ZnS and ZnO nanocrystals.

**Figure 3:**
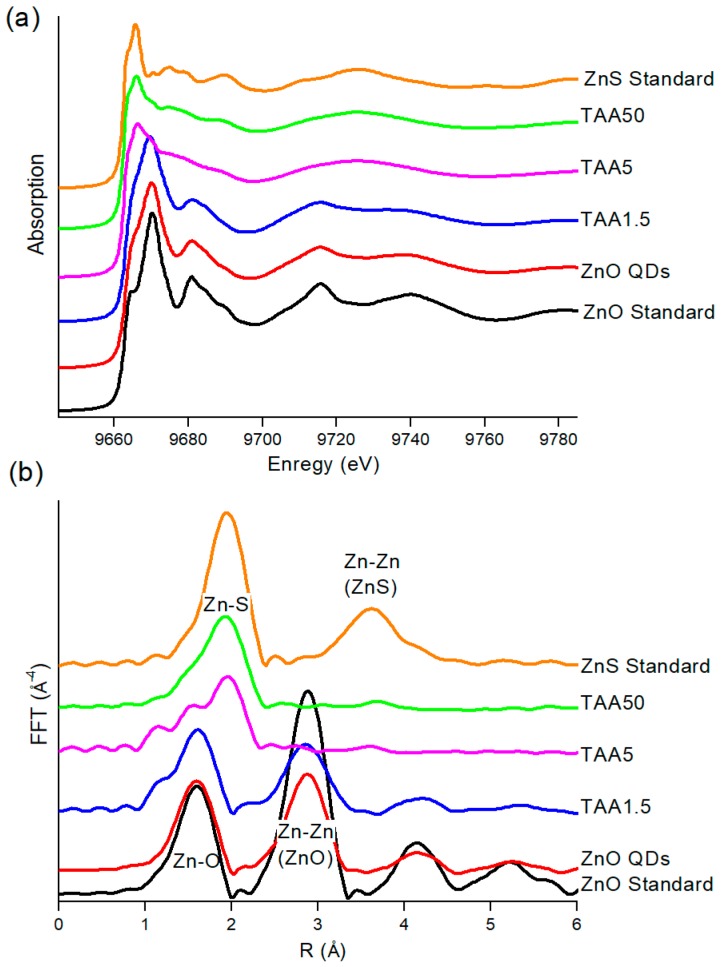
(**a**) X-ray absorption near edge structure (XANES) spectra; and (**b**) Fourier Transforms of extended X-ray absorption fine structure (EXAFS) spectra recorded for the different samples and ZnO and ZnS standard references.

Figure 3a,b displays the X-ray absorption near edge structure (XANES) spectra and Fourier Transforms (FT) of EXAFS spectra recorded for the different samples and compared to the standard references. Significant differences in XANES shape and white line positions can be observed between the standard ZnO and ZnS references. The FT of ZnO EXAFS spectrum is characterized by two main contributions, the first one corresponding to the oxygen tetrahedral coordination shell at 1.96 Å and a second one related to the zinc second next neighbors at 3.23 Å [38], whereas ZnS presents essentially a first tetrahedral sulfur coordination shell at 2.34 Å and a broad contribution with low intensity compared to ZnO corresponding to the zinc second next neighbors at 3.82 Å [39].

TAA1.5 sample displays a XANES spectrum very similar to those recorded for ZnO QDs and ZnO standard. This feature indicates that this sample consists mainly of ZnO nanoparticles. Conversely, the sample prepared with the highest TAA amount (50 mM) displays a XANES spectrum closer to that of ZnS standard. This finding evidences that the large amount of sulfur ions in the medium favored the formation of nanoparticles presenting local order arrangement comparable to ZnS.

FT spectra fully support the conclusions drawn from the XANES spectra. Samples with ZnO QD characteristics (TAA1.5) and those with ZnS features (TAA50) show FT peaks located at the same positions as the respective standards. The FT spectrum of the sample prepared with intermediate TAA concentration (5 mM) is characterized by the double maximum of the first contribution, revealing the simultaneous presence of ZnO and ZnS, in agreement with XRD and HRTEM results.

Further insights into the structural features of the samples were obtained by the least-square fitting procedures of the first coordination shells. The so obtained structural parameters are gathered in Table 1. As expected, TAA1.5 is characterized by 3.6 ± 0.2 oxygen atoms at 1.99 ± 0.01 Å. This confirms that only ZnO QDs are observed for the lowest TAA concentration. TAA50 can be described by a coordination shell made of 3.0 ± 0.2 sulfur atoms at 2.34 ± 0.01 Å and 1.2 ± 0.1 oxygen atoms at 2.02 ± 0.01 Å. Of note, no contribution at larger distances is observed. The XANES spectrum of TAA50 is satisfactorily fitted by a linear combination of ZnO QD and ZnS standard spectra, in the ~25:75% proportion. This composition is in perfect agreement with the oxygen and sulfur coordination numbers reported in Table 1, suggesting that this sample is a mixture of both QDs: ~25% of ZnO QDs and 75% of ZnS QDs. The lack of medium range order can be explained by the small size of ZnS QDs (~1.4 nm), compared to that of ZnO QDs (~5.3 nm), resulting in no detectable contribution of Zn-Zn second neighbors to the FT spectra.

In the case of TAA5, the relative proportions of ZnO QDs and ZnS QDs deduced from LCF of the XANES spectrum are 37% and 63%, respectively, which should lead to about 1.5 oxygen atoms and 2.5 sulfur atoms in the first coordination shell of Zn. These values are very close from those retrieved from the least-square fitting procedures, suggesting a first coordination shell around Zn composed of 2.0 ± 0.6 oxygen atoms at 2.00 ± 0.02 Å and 2.2 ± 1.0 sulfur atoms at 2.34 ± Å. The slight increase of the number of O atoms, 2.0 instead of 1.5, observed could be associated to the formation of the core–shell structure.

### 3.2. Time-Resolved Study of the Nanoparticle Synthesis

The formation of the QDs was first studied by UV-Vis absorption spectroscopy. Figure S1a in the Supplementary Materials shows the UV-vis spectra of ZnO QDs, ZnS QDs and a mixture of ZnO and ZnS QDs and Figure S1b presents the UV-vis spectrum of the ethanolic solution of TAA. The excitonic peaks at about 290 nm and 350 nm are characteristic of ZnS QDs [40] and ZnO QDs [26], respectively, and the band around 266 nm is the fingerprint of TAA. The decrease of the TAA absorption band reflects the release of S^−2^ ions into the solution. QDs are characterized by a decreasing band gap, and thus a red-shift of their excitonic absorption, with increasing QD size. The radius of ZnO QDs (R_UV-vis_) could therefore be determined from the absorption spectra using the effective mass model derived by Brus.

**Figure 4:**
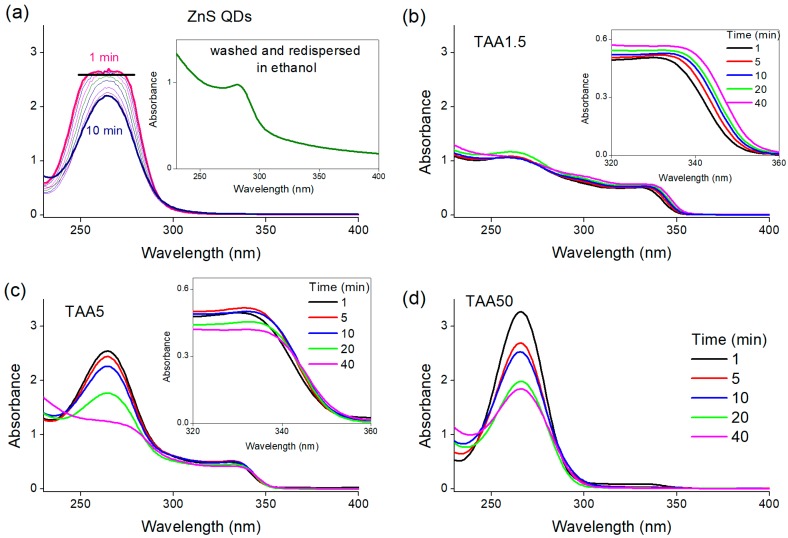
Selected UV-vis spectra measured at the indicated reaction times (min) for: (**a**) ZnS quantum dots (QDs); and different thioacetamide (TAA) concentrations: (**b**) 1.5 mM; (**c**) 5 mM; and (**d**) 50 mM. The inset in (**a**) shows the spectrum of the ZnS QDs washed and redispersed in ethanol. The insets in (b) and (c) show a zoom of the ZnO excitonic peaks at the indicated reaction times (min) for TAA1.5 and TAA5, respectively.

**Figure 5:**
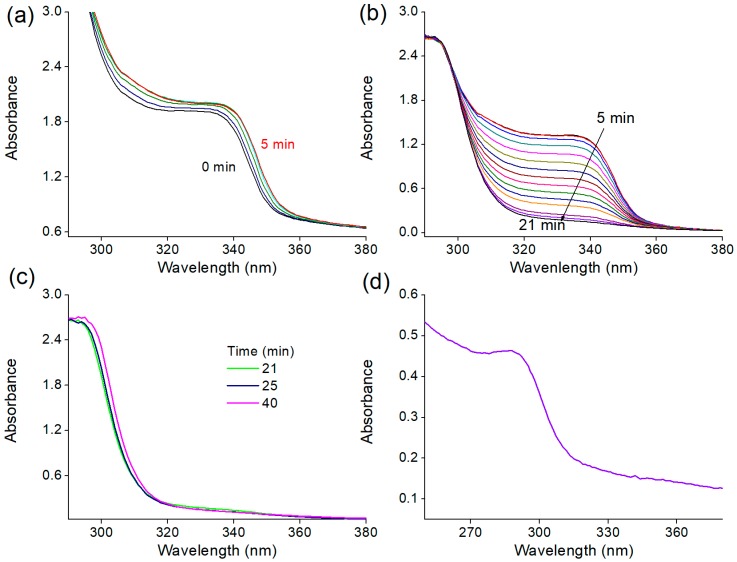
Selected UV-vis spectra of TAA50 reaction measured at the indicated reaction times (min): (**a**) 1–5; (**b**) 5–21; and (**c**) 21–40; and (**d**) the spectrum of the final product washed and redispersed in ethanol.

UV-vis absorption spectra measured at 1, 5, 10, 20 and 40 min of reaction for the different TAA concentrations are shown in Figure 4. ZnS QDs formation is also show in Figure 4a for comparison to the other reactions. The inset in Figure 4a shows the spectrum of the ZnS washed and redispersed in ethanol, which exhibited the excitonic peak at about 290 nm characteristic of ZnS QDs. For TAA50, the reaction was also monitored in situ and time-resolved UV-vis spectra are shown in Figure 5.

At the lower TAA concentration, we observe a red-shift of the excitonic peak of ZnO corresponding to a growth of about 0.3 nm. Moreover, the absence of a ZnS excitonic peak confirms the sole presence of ZnO nanocrystals. 

For TAA5 we do not observe any displacement of the excitonic peak of ZnO as a function of time, indicating that the size of the ZnO nanoparticles remains constant. This behavior might be explained by the formation of a ZnS shell around the ZnO core, preventing the growth of the latter. On the other hand, the absorbance varied along the time. It increased slightly in the first minutes of the reaction, reflecting an increase in the number of ZnO QDs (inset of Figure 4c). It can be assumed that the nucleation of new ZnO QDs resulted from the hydrolysis and condensation of the precursor still present in the suspension. After about 5 min, the opposite trend was observed: the absorbance decreased along time. We can suggest that some of the ZnO QDs could be converted into ZnS. At the end of the reaction, both excitonic peaks of ZnO and ZnS were observed.

For TAA50, the ZnO peak present at the beginning of the reaction slowly decreases to finally vanish as the reaction proceeds. This reaction was monitored in situ and the Figure 5 presents selected UV-vis spectra of different reaction times (min) (Figure 5a) 1–5, (Figure 5b) 5–21, and (Figure 5c) 21–40, as well as (Figure 5d) the spectrum of the final product washed and redispersed in ethanol. From 1 to 5 min (Figure 5a), a red-shift accompanied by the increase of absorbance intensity is observed, demonstrating a slight increase of the size of ZnO QDs and the increase of the number of ZnO nanoparticles, as observed for TAA5. From 5 to 21 min of reaction (Figure 5b), the absorbance intensity of the ZnO excitonic peak decreases. This can be interpreted as the consumption of the ZnO nanoparticles with the simultaneous formation of ZnS. From 23 to 40 min (Figure 5c), the ZnO excitonic peak is absent and a red-shift of the peak around 320 nm is observed demonstrating the growth of ZnS QDs. To confirm the formation of ZnS nanoparticles, the TAA50 reaction product was washed in order to remove the unreacted TAA, which dominates the absorption spectrum in this wavelength range (Figure 4c), and re-suspended in ethanol before UV spectroscopy analysis. As expected, the peak around 290 nm fully confirms the formation of ZnS nanoparticles as the main phase (Figure 5d).

**Figure 6:**
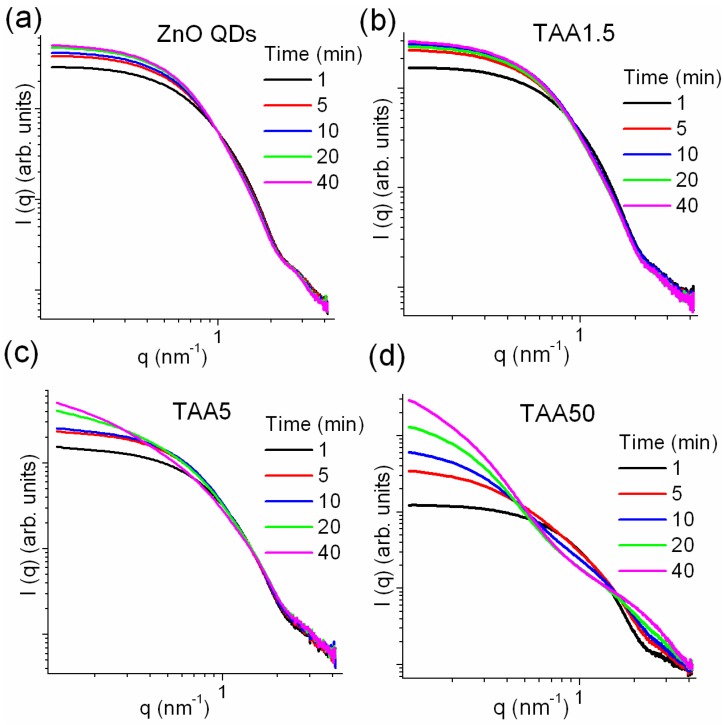
Selected small angle X-ray scattering (SAXS) profiles recorded in situ at the indicated reaction times (min) for: (**a**) ZnO quantum dots (QDs); and for different thioacetamide (TAA) concentrations: (**b**) 1.5 mM; (**c**) 5 mM; and (**d**) 50 mM.

The growth of the different populations of nanoparticles was further evidenced by time-resolved SAXS patterns recorded from the beginning of the reactions. The reactional solution was injected in the sample holder and kept at 60 °C and the reaction was monitored during 40 min. Figure 6 presents the log–log plot of selected SAXS curves measured at increasing times of reaction for the different concentrations of TAA. The SAXS curves of ZnO QDs ([TAA] = 0, Figure 6a) display a plateau at low *q*-range (Guinier region) and an asymptotic linear decrease in the high-*q* range (Porod region), characteristic of a dilute suspension of nanoparticles. In the Guinier region, the scattered intensity can be approximated by *I*(*q*) = *I*(0) exp(−*R*g^2^*q*^2^/3) where *R*g is the radius of gyration (Guinier radius) of the particles or aggregates and *I*(0) is the limit of *I*(*q*) when *q* → 0, given by *I*(0) = *N* × (*ρ*_p_ − *ρ*_s_)2 × *V*^2^, where *N* is the particle number density, *ρ*_p_ and ρ_s_ are the average electron densities of the particles and the solution, respectively, and *V* is the volume of the particle [41]. The gradual shift of curves toward lower q values reflects the increase of the mean nanoparticle size while the nucleation of new nanoparticles induces an increase in *I*(0). A similar behavior was observed for TAA1.5 and TAA5.

**Figure 7:**
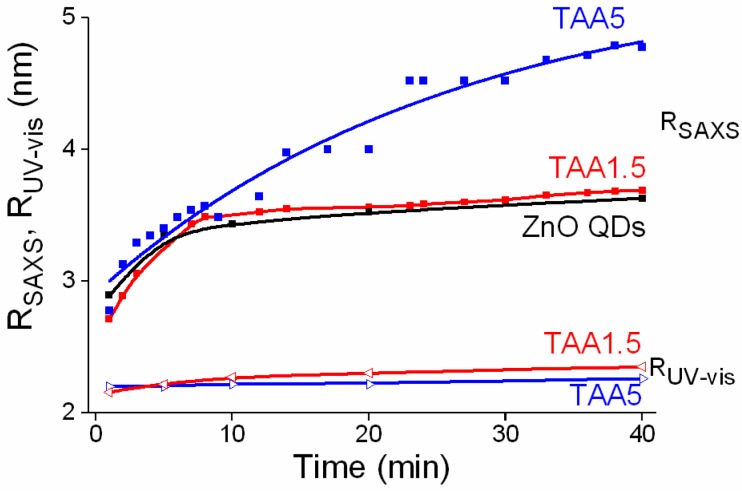
Time evolution of the quantum dots (QDs) average radii (*R*_SAXS_) deduced from *R*g values (*R*_SAXS_ = (5/3)^1/2^ *R*g for a spherical NP) of colloidal particles formed using 0 (ZnO QDs), 1.5 and 5 mM thioacetamide (TAA) concentrations and the *R*_UV-vis_ calculated by Brus equation from UV spectra of TAA1.5 and TAA5. Solid lines are guides for the eye.

Figure 7 gathers the time evolution of the QD average radii (*R*_SAXS_) deduced from *R*g values (*R*_SAXS_ = (5/3)^1/2^ *R*g for a spherical NP) of colloidal particles formed using 0 (ZnO QDs), 1.5 and 5 mM TAA concentrations. For TAA concentration equal to 1.5 mM the evolution of *R*_SAXS_ is almost identical to that of ZnO QD *R*_SAXS_ ([TAA] = 0.0 mM). TAA had a negligible effect on the growth of colloids. *R*_SAXS_ of TAA5 displays a similar evolution until 10 min. After that, *R*_SAXS_ increases from 3.5 to 4.8 nm at the end of the reaction. The comparison of *R*_UV-vis_, which remains constant in the course of the reaction, and *R*_SAXS_ suggest that the *R*_SAXS_ increase is related to aggregation of nanoparticles and/or to the growth of ZnO/ZnS heterostructures: ZnS shells could form on ZnO, yielding larger scattering objects.

The SAXS profiles recorded during synthesis of TAA50 samples are markedly different (Figure 6d): the first curves, characterized by a Guinier regime and an asymptotic linear decrease at high *q*, are typical of a single population of diluted nanoparticles, whereas the curves recorded later show two Gaussian decays, suggesting the presence of two populations of nanoparticles having different average sizes.

**Figure 8:**
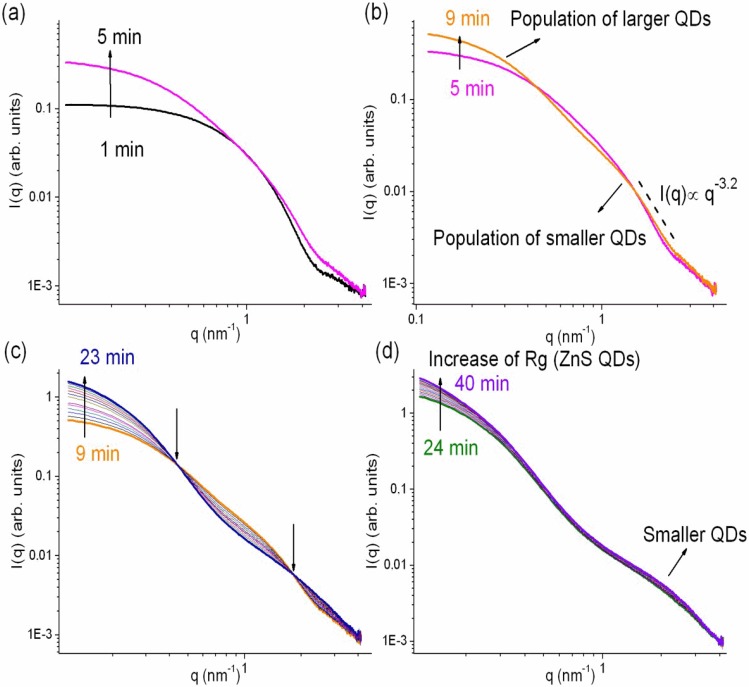
Small-angle X-ray scattering (SAXS) curves recorded during TAA50 reaction at different time intervals: (**a**) 1–5 min; (**b**) 5–9 min; (**c**) 9–23 min; and (**d**) 24–40 min. In (**c**) the two arrows at *q*1 = 0.44 nm^−1^ and at *q*2 = 1.78 nm^-1^ indicate the two isobestic points.

We highlight in Figure 8 four time intervals characteristic of the different evolution steps of TAA50 SAXS curves between: (a) 1–5 min; (b) 5–9 min; (c) 9–23 min; and (d) 24–40 min.

At the early reaction time (Figure 8a), the mean QD size increases, in agreement with the red-shift of the ZnO excitonic peak. The main modification in the shape of the SAXS curves observed between ~5 and 9 min (Figure 8b) is then the emergence of the second Gaussian decays. The slope at high *q* range (−3.2) corresponds to a diffuse interface, possibly due to the formation of a shell of variable composition or to localized etching of the nanoparticles resulting in a rough surface. After this step, all the SAXS curves clearly display two Gaussian decays. In the intermediate period (Figure 8c: 9 to 23 min), the curves present a double crossover at *q*1 = 0.44 nm^−1^ and at *q*2 = 1.78 nm^−1^, characterizing two isobestic points. The existence of these isobestic points implies that at least two equilibrium states describe the nanostructural transformation occurring from 9 to 23 min. One population of scatters grows at the expense of the other one. In the last step (Figure 8d), the largest nanoparticles continue to grow.

**Figure 9:**
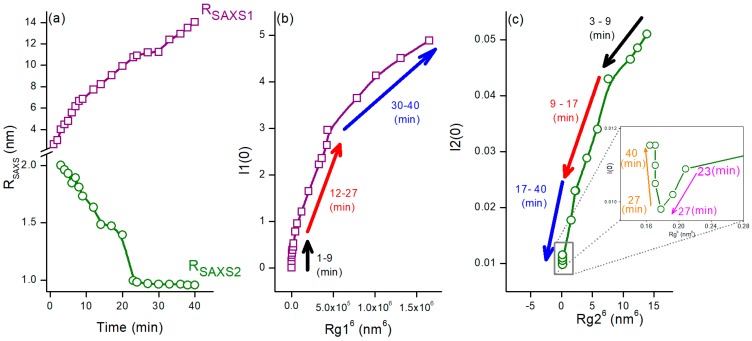
(**a**) Time evolution of the average radii *R*_SAXS1_ and *R*_SAXS2_ deduced from *R*g values (*R*_SAXS_ = (5/3)^1/2^ *R*g for a spherical NP); (**b**) *I*1(0) versus *R*g1^6^ plot; and (**c**) *I*2(0) versus *R*g2^6^ plot of TAA50, where *I*(0) is the limit of *I*(*q*) when *q* → 0.

The analysis of the SAXS experimental results for TAA50 was therefore carried out considering two populations (denoted as 1 and 2) of nanoparticles; SAXS data were satisfactorily fitted with the sum of two distributions, characterized by the mean average radii of gyration *R*g1 and *R*g2 (see Supplementary Materials). Figure 9a gathers the time evolution of *R*_SAXS1_ and *R*_SAXS2_ deduced from *R*g1 and *R*g2 values, respectively (*R*_SAXS_ = (5/3)^1/2^ *R*g for a spherical NP), Figure 9b the variation of *I*1(0) versus *R*g1^6^ and Figure 9c *I*2(0) versus *R*g2^6^. The increase in *I*1(0) in the first minutes while *R*g1^6^ remains almost constant highlights the nucleation of new nanoparticles. Then, as the reaction progresses, the evolution of *I*1(0) suggests the growth and aggregation of existing nanoparticles (Figure 9b). On the other hand, the size *R*g2 of the nanoparticles belonging to the second population slightly decreases with time until about 27 min (Figure 9c).

The large objects seen in the low *q*-range at the end of the synthesis are likely aggregates of small ZnS crystals. However, it is less clear whether the second population corresponds to precursor clusters, to ZnO QDs, that progressively dissolve, or to ZnS QDs that nucleate.

The above results can be summarized as follows: 

TAA1.5: ZnO QDs were almost exclusively formed, as shown by UV-vis absorption, XAS and XRD. ZnS did not form, likely because the available amount of sulfur was too small.

TAA5: All of the characterization techniques evidenced the coexistence of ZnO and ZnS species and/or the formation of heterostructures. Taken together, UV-vis absorption spectroscopy and SAXS experiments indicated that ZnO nanoparticle growth or dissolution might be hindered by the formation of a ZnS shell. Although thin shells cannot easily be evidenced by HRTEM, some HRTEM images displayed a ZnS crystal attached on a ZnO nanoparticle. The weak UV emission and the reduced green emission, as compared to the photoluminescence of ZnO QDs and TAA1.5, also supported the formation of ZnO/ZnS heterostructures (see Supplementary Materials). However, the full coverage of the ZnO core with a dense ZnS shell would enhance the UV luminescence more strongly. This suggests the formation of a discontinuous shell, likely made of small ZnS nanocrystallites. Regarding the visible green emission, originating mainly from surface defects such as oxygen vacancies, it could be quenched by even a small amount of sulfur ions on the surface [10,17,20,21,42].

TAA50: ZnS QDs were mainly formed as shown by XRD, XAS and UV-vis spectroscopy. Small QDs formed large aggregates. In addition to Zn precursor remaining in solution at the beginning of the reaction (~80%), Zn ions from the surface of the ZnO QDs could easily react with the abundant S ions to form ZnS.

The concentration of TAA, used as sulfur source, is the main parameter controlling the conversion of ZnO to ZnS. However, the reaction medium is complex and many chemical reactions can take place. Indeed, some water is brought into the ethanolic precursor solution by the dissolution of ${\mathrm{ZnAc}}_{2}\cdot {2H}_{2}O$ and LiOH·H_2_O. When the precursor is prepared, acetate moieties are released, which are able to form acetic acid or to react with ethanol to yield ester and additional water [38,43]. In the presence of water, TAA can decompose to release acetamide and S ions, leading to the formation of ZnS. The progressive hydrolysis of TAA with time could sustain the growth of ZnS in the TAA5 and TAA50 systems. Of note, for the TAA50 system, the [Zn]/[S] ratio is 1. Therefore, a full conversion of ZnO into ZnS could be expected according to their respective solubility constants. However, XAS unambiguously demonstrated the presence of O atoms near Zn atoms. As a tentative explanation, we might suggest that completion of the reaction would have needed more time. Indeed, when ZnS formation is initiated on the ZnO surface, further conversion reaction requires diffusion of ionic species (inward S diffusion and outward O diffusion).

## 4. Conclusions

Depending on synthesis conditions, ZnS formation could take place via direct crystal growth involving Zn ions remaining in solution and S ions provided by TAA and/or interfacial sulfidation of ZnO. The influence of TAA concentration was evidenced. ZnO and ZnS phases were mainly obtained at low and high TAA concentrations, respectively. At intermediate TAA concentrations, we observed the formation of ZnO/ZnS heterostructures. The reaction steps were monitored in situ by SAXS, and UV-vis spectroscopy; the final products were further characterized by XRD, HR-TEM and XAS. This detailed study contributes to the understanding of the mechanisms underlying the formation of ZnO/ZnS heterostructures via a sol-gel route.

## Figures and Tables

**Table 1:** Extended X-ray absorption fine structure (EXAFS) structural parameters for the first Zinc coordination sphere of ZnO and ZnS, and the percentages of ZnO and ZnS deduced from the Linear Combination Fittings (LCF) of X-ray absorption near edge structure (XANES) spectra.

Sample	N_Zn-O_	R_Zn-O_	σ^2^_Zn-O_ (Å^2^)	N_Zn-S_	R_Zn-S_	σ^2^_Zn-S_ (Å^2^)	R_factor_	LCF	
								% ZnO	% ZnS
ZnO Standard	4	1.97	0.004 ± 0.002				0.0003		
ZnO QDs	3.7 ± 0.4	1.97	0.005 ± 0.001				0.0014		
TAA1.5	3.6 ± 0.2	1.99	0.006 ± 0.001				0.0003	98	2
TAA5	2.0 ± 0.6	2	0.007 ± 0.005	2.2 ± 1.0	2.34	0.007 ± 0.005	0.0027	37	63
TAA50	1.2 ± 0.1	2.02	0.008 ± 0.001	3.0 ± 0.2	2.34	0.008 ± 0.001	0.0001	23	77
ZnS Standard				4	2.34	0.006 ± 0.001	0.0004		

## Supplementary Materials

The following are available online at http://www.mdpi.com/2079-4991/8/2/55/s1, Figure S1: UV-vis spectra of: (a) ZnO QDs, ZnS QDs and the mixture of ZnO and ZnS QDs as-synthesized colloidal suspensions; and (b) ethanolic solution of TAA, Figure S2: Example of SAXS curve of TAA50 at the end of the reaction (about 36 min) fitted with the form factors of two populations of homogeneous spheres displaying Lognormal radius distribution using SASfit software, Figure S3: Photoluminescence emission spectra of ZnO QDs, TAA1.5 and TAA5 excited at 353 nm, and TAA50 and ZnS QDs excited at 302 nm.
