# Supplementary material for: The Critical Role of Thioacetamide Concentration in the Formation of ZnO/ZnS Heterostructures by Sol-Gel Process
Source: Nanomaterials (Basel). 2018 Jan 23;8(2):55. doi: 10.3390/nano8020055 (PMC5853688; doi:10.3390/nano8020055)
Supplement: Supplementary file 1 [file nanomaterials-08-00055-s001.pdf]

Supplementary Materials

The critical role of thioacetamide concentration in the formation of ZnO/ZnS heterostructures by sol-gel process

Eloísa Berbel Manaia [1,2,*], Renata Cristina Kiatkoski Kaminski [3], Bruno Leonardo Caetano [1], Marina Magnani [4], Florian Meneau [5], Amélie Rochet [5], Celso Valentim Santilli [4], Valérie Briois [6], Claudie Bourgaux [2] and Leila Aparecida Chiavacci [1,*]

1 Department of Drugs and Medicines, School of Pharmaceutical Sciences, São Paulo State University (UNESP), Araraquara, São Paulo 14800-903, Brazil; caetano@fcfar.unesp.br
2 Institut Galien, University Paris-Sud, The National Center for Scientific Research (CNRS), UMR 8612, F-92296 Châtenay-Malabry, France; claudie.bourgaux@u-psud.fr
3 Department of Chemistry, Sergipe Federal University—Campus Itabaiana, Av. Vereador Olimpio Grande, s/n—Itabaiana, SE 49506-036, Brazil; re_kaminski@hotmail.com
4 Chemistry Institute of São Paulo State University—UNESP, Prof. Francisco Degni Street, 55, Araraquara, São Paulo 14800-060, Brazil; marina@iq.unesp.br (M.M.); santilli@iq.unesp.br (C.V.S.)
5 Brazilian Synchrotron Light Laboratory (LNLS), Brazilian Center for Research in Energy and Materials (CNPEM), 13083-970 São Paulo, Brazil; florian.meneau@lnls.br (F.M.); amelie.rochet@lnls.br (A.R.)
6 *Synchrotron* Optimized Light Source of Intermediate Energy to LURE (*SOLEIL), L'Orme des Merisiers, BP48, Saint Aubin, 91192 Gif-sur Yvette, France;* valerie.briois@synchrotron-soleil.fr
* Correspondence: manaiaeb@fcfar.unesp.br (E.B.M.); leila@fcfar.unesp.br (L.A.C.); Tel.: +55-16-3301-4686 (E.B.M.); +55-16-3301-6966 (L.A.C.)

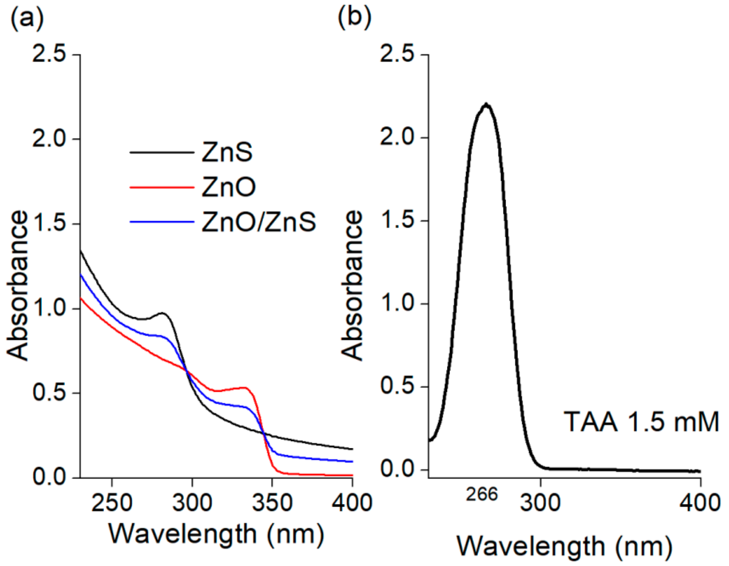

Figure S1. UV-vis spectra of (**a**) ZnO QDs, ZnS QDs and the mixture of ZnO and ZnS QDs as-synthesized colloidal suspensions and (**b**) ethanolic solution of TAA.

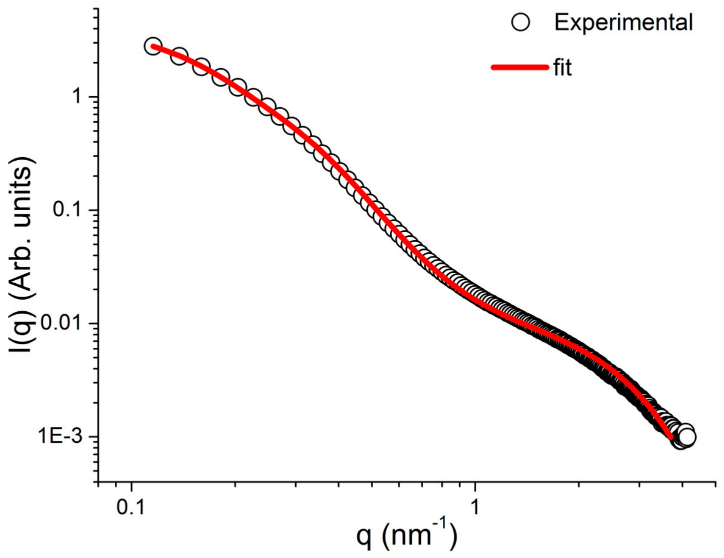

Figure S2. Example of SAXS curve of TAA50 at the end of the reaction (about 36 min) fitted with the form factors of two populations of homogeneous spheres displaying Lognormal radius distribution using SASfit software.

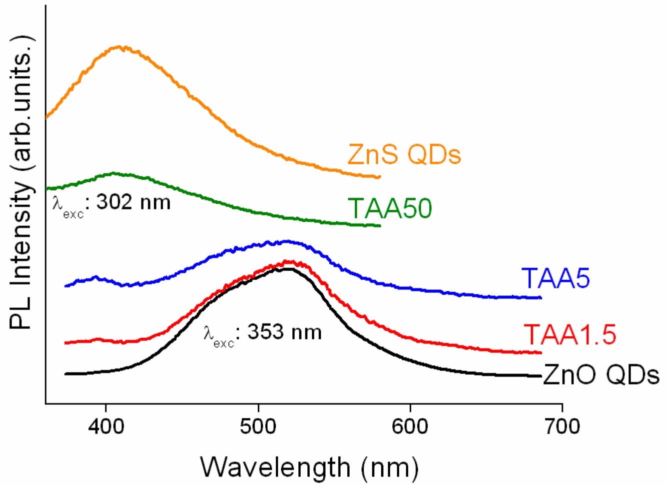

Figure S3. Photoluminescence emission spectra of ZnO QDs, TAA1.5 and TAA5 excited at 353 nm and TAA50 and ZnS QDs excited at 302 nm.

ZnO QDs can present typically two main emission contributions: (i) the exciton emission (photo-generated electrons recombine with the holes in the valence band) in the UV range, and (ii) a broad visible luminescence (originated from defects such as oxygen vacancies) usually around 500 nm. In our study, it was observed a decrease in the visible emission and a weak increase in the UV emssion for the TAA5 compared to TAA1.5 and ZnO QDs. During the sulfidation process, the formation of a discontinuous shell, likely made of small ZnS nanocrystallites, may give rise to the decrease in oxygen vacancies on ZnO QDs surface, resulting in the decrease in visible emission. The small increase in UV emission can be explained by the deposition of a higher bandgap discontinuous shell on the lower bandgap core, which passivates the surface of the ZnO core [1–4].

References

1. Verma, P.; Pandey, A.C.; Bhargava, R.N. Synthesis and characterisation: Zinc oxide–sulfide nanocomposites. *Physica B Condens Matter* **2009**, *404*, 3894–3897.
2. Sharma, S.; Chawla, S. Enhanced UV emission in ZnO/ZnS core shell nanoparticles prepared by epitaxial growth in solution. *Electronic Materials Letters* **2013**, *9*, 267–271.
3. Rabani J. Sandwich colloids of zinc oxide and zinc sulfide in aqueous solutions. *J Phys Chem* **1989**, *93*, 7707–7713.
4. Shuai, X. M.; Shen, W. Z. A Facile Chemical Conversion Synthesis of ZnO/ZnS Core/Shell Nanorods and Diverse Metal Sulfide Nanotubes. *J Phys Chem C Nanomater Interfaces* **2011**, *115*, 6415–6422.
